# Two new pseudoscorpion species (Pseudoscorpiones, Chthoniidae, Cheiridiidae) from the Tonga Islands, Polynesia, with a redescription of the genus *Nesocheiridium*

**DOI:** 10.3897/zookeys.927.49351

**Published:** 2020-04-16

**Authors:** Katarína Krajčovičová, Aleksandr Vladimirovich Matyukhin, Jana Christophoryová

**Affiliations:** 1 Department of Zoology, Faculty of Natural Sciences, Comenius University, Mlynská dolina, Ilkovičova 6, SK–842 15 Bratislava, Slovakia Comenius University Bratislava Slovakia; 2 Institute of Ecology and Evolution, Severtsov Russian Academy of Sciences, Federal State Institution of Science, Leninsky pr. 33, 117 071 Moscow, Russia Institute of Ecology and Evolution, Severtsov Russian Academy of Sciences Moscow Russia

**Keywords:** Endemism, *
Nesocheiridium
*, Oceania, taxonomy, *
Tyrannochthonius
*

## Abstract

The genera *Tyrannochthonius* Chamberlin, 1929 and *Nesocheiridium* Beier, 1957 are recorded from the Tonga Islands, Polynesia, for the first time. *Tyrannochthonius eua***sp. nov.** is described from the island of Eua. *Nesocheiridium onevai***sp. nov.** is described from the island of Onevai. This is the first discovery of a representative of the genus *Nesocheiridium* in more than 60 years. The holotype of the type species, *Nesocheiridium stellatum* Beier, 1957, is redescribed, allowing a better understanding of this poorly known genus. The genus *Nesocheiridium* is diagnosed by the following combination of characters: integument coarsely granulate, dorsally granulo-reticulate; vestitural setae either relatively long, with a leaf-like outline, or arcuate with a small spine; cucullus short; only 10 abdominal tergites visible in dorsal view; cheliceral rallum of four blades; venom apparatus present in both chelal fingers; fixed chelal finger with granulate swelling mesally and seven trichobothria; trichobothria *ib* and *ist* located distad of granulate swelling; *eb* and *esb* situated close together at the base of the finger; moveable chelal finger with two trichobothria.

## Introduction

Polynesia is a subregion of Oceania, comprising more than a thousand islands spread across the central and southern Pacific Ocean. The small size of the islands and their isolation promote strong evolutionary selection (Filin and Ziv 2004) and high endemism of the fauna (Udvardy 1965). During an expedition to collect invertebrates in Oceania in 1980, a few pseudoscorpions were collected on the Tonga Islands. The Kingdom of Tonga comprises 169 islands, stretching approximately 800 km in a north-south line in Polynesia, flanked by Fiji to the northwest and Samoa to the northeast. Tonga, like much of Polynesia, is poorly known in terms of its pseudoscorpion fauna. Except for New Zealand (Harvey 2013), only a few works have dealt with the pseudoscorpions of this region (With 1907; Kästner 1927; Beier 1932, 1940; Chamberlin 1939a, b; Muchmore 1979, 1983, 1989, 1993, 2000; Harvey 2000). Only a single species, *Geogarypus longidigitatus* (Rainbow, 1897), had been recorded from the Tonga Islands before now (Harvey 2000). Two species are added here, belonging to the genera *Tyrannochthonius* Chamberlin, 1929 and *Nesocheiridium* Beier, 1957.

The genus *Tyrannochthonius* is widely distributed in tropical and subtropical regions of the world. It is one of the largest chthoniid genera, with 130 described species. Most of these have restricted distributions, known from only a few locations. The available data indicate a tendency for short-range endemism of its species (Edward and Harvey 2008; Harvey 2013). In Polynesia, *Tyrannochthonius* species have only been recorded from Hawaii (Muchmore 1983, 1989, 1993, 2000) and New Zealand (Chamberlin 1929; Beier 1966, 1967, 1976). The Tongan specimen belongs to a new species, which is described here.

The genus *Nesocheiridium* was erected in the Cheiridiidae by Beier (1957), with *Nesocheiridium stellatum* Beier, 1957 as its only included species. Until now, the holotype of *N. stellatum*, from Saipan, Marianna Islands, Micronesia, has been the only known specimen of the genus. A re-examination of that species and the new species described here allow a better characterization of the genus.

## Methods

All specimens examined for this study had been preserved in 75% ethanol. They were studied as temporary slide mounts, prepared by immersing the specimens in lactic acid for clearing. After study, they were rinsed in water and returned to 75% ethanol, with the dissected portions placed in microvials.

Morphological and morphometric analyses were performed using a Leica DM1000 compound microscope with an ICC50 Camera Module (LAS EZ application, 1.8.0). Measurements were taken from digital images using the AxioVision 40LE application. Reference points for measurements follow Chamberlin (1931), except that the pedicel was included in the measurements of the lengths of the chela and chelal hand. Drawings were made using a Leica DM1000 drawing tube. Digital photographs of new species (Figs 2, 4) were taken using a Canon EOS 5D camera attached to a Zeiss Axio Zoom.V16 stereomicroscope. Image stacks were produced manually, combined using Zerene Stacker software, and edited with Adobe Photoshop CC. Photographs of *N. stellatum* were taken at the Collaborative Invertebrate Laboratory, Field Museum, Chicago, USA (**FMNH**) using a Digital Microptics system consisting of a Nikon D5100 camera, a flash lighting system, P-51 Camlift with controller and software including Base plate, on a computer workstation.

Terminology follows Chamberlin (1931), except for the naming of the palpal and pedal segments (Harvey 1992) and the use of the terms rallum (Judson 2007) and duplex trichobothria (Judson 2018). Trichobothrial homologies follow Harvey (1992).

The types of new species are deposited in the zoological collections of the Naturhistorisches Museum Wien, Austria (**NHMW**).

## Results

### Chthoniidae Daday, 1889

#### Tyrannochthonius

Taxon classificationAnimaliaPseudoscorpionesChthoniidae

Chamberlin, 1929

FD22B305-E341-5612-8FD3-8E77A0D262FB

##### Diagnosis.

See Edward and Harvey (2008).

#### Tyrannochthonius eua sp. nov.

Taxon classificationAnimaliaPseudoscorpionesChthoniidae

BE47A517-201B-557F-B297-A5EB8B63071D

http://zoobank.org/F65825EA-F0DC-4D86-92E5-8B7D67C38383

Figs 1 , 2 , 3

##### Material examined.

***Holotype***: Polynesia • ♂; Tonga, Eua [-21.387, -174.930]; 215 m a.s.l.; 11 Jul. 1980; Galina Fedorovna Kurcheva leg.; moss; **NHMW** 29197.

##### Description.

***Adult male*** (Figs 2, 3). ***Carapace*** (Fig. 3A): 0.97 × longer than broad; with four corneate eyes; epistome present, triangular; with 18 setae arranged 6: 4: 4: 2: 2; without furrows; with two pairs of small lyrifissures, first pair situated in ocular row, second pair situated lateral to setae of posterior row. ***Coxae*** (Fig. 3B): coxa I with rounded apical projection, not bearing microsetae; chaetotaxy of coxae (Fig. 3B): palpal coxae 3; pedal coxae I 3, II 4, III 5, IV 5. Coxa II with eight terminally incised spines, set in an oblique row (Fig. 3B, C). Intercoxal tubercle absent. ***Chelicera*** (Fig. 3D): 1.53 × longer than broad; five setae on hand, all acuminate; moveable finger with one medial seta; fixed finger with 11, moveable finger with nine teeth; one ventral and two dorsal lyrifissures on hand; galea absent; serrula exterior with 15 blades; rallum consisting of seven bipinnate blades. ***Pedipalp*** (Fig. 3E): all setae acuminate, femur setal formula: 5: 2: 1: 3: 5; trochanter 1.44 ×, femur 4.11 ×, patella 2.11 ×, chela 5.18 ×, hand 2.18 × longer than broad. Hand without spine-like seta, dorsal surface with a single row of five chemosensory setae between trichobothria *esb* and *ib*/*isb*; hand and fixed chelal finger together with eight trichobothria, moveable chelal finger with four trichobothria; *ib* and *isb* close together, submedially on dorsum of chelal hand; *eb* and *esb* close together, at base of fixed finger; *ist* distal to *eb* and *esb*; *it* and *est* less than one areolar diameter apart, *it* slightly distal to *est*; *et* near tip of finger; trichobothrium *st* of moveable finger sub-basally; *sb* slightly closer to *st* than to *b*; *b* and *t* subdistally, *t* at same level as *it*; *b* slightly basal to *est.* Chelal teeth heterodentate: fixed finger with three small teeth followed by 17 large, erect, well-spaced teeth, decreasing in size towards base, distally alternating with six small intercalary teeth; moveable finger with nine large, erect, well-spaced teeth, without intercalary teeth. ***Opisthosoma***: tergites and sternites undivided; setae uniseriate and acuminate. Tergal chaetotaxy I–IX: 4: 4: 4: 4: 4: 5: 6: 6: 6. Sternal chaetotaxy II–IX: 10: 28: 15: 10: 10: 8: 8: 8 (Fig. 3F). Sternal lyrifissures II–IX: 2: 2: 0: 0: 0: 0: 0: 0. Genitalia: sternite III with narrow V-shaped opening. Genitalia not studied in detail. ***Leg I***: trochanter 1.13 ×, femur 4.40 ×, patella 2.75 ×, tibia 3.25 ×, tarsus 5.67 × deeper than broad. ***Leg IV***: trochanter 1.22 ×, femoropatella 1.88 ×, tibia 3.43 ×, metatarsus 2.00 ×, tarsus 6.67 × deeper than broad. Tactile seta present on metatarsus of leg IV; arolium slightly shorter than claws, not divided; claws simple.

**Figure 1.**
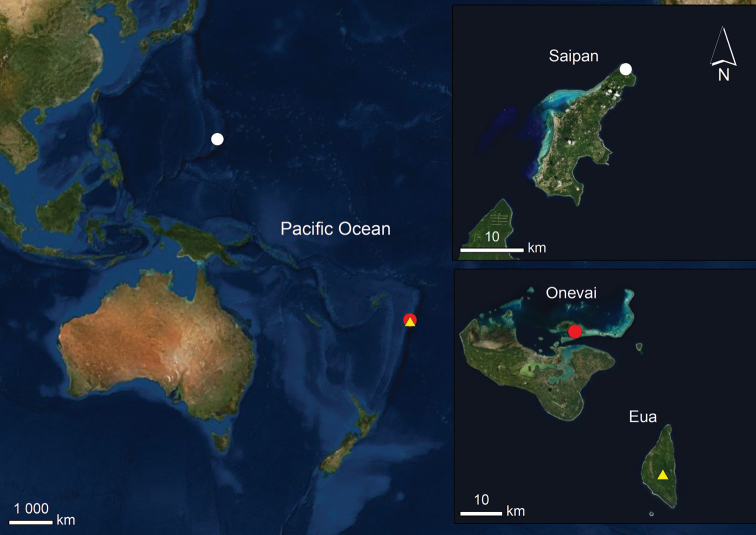
Distribution of the studied species: *Tyrannochthonius eua* sp. nov. (yellow triangle), *Nesocheiridium stellatum* (white circle), *N. onevai* sp. nov. (red circle).

**Figure 2.**
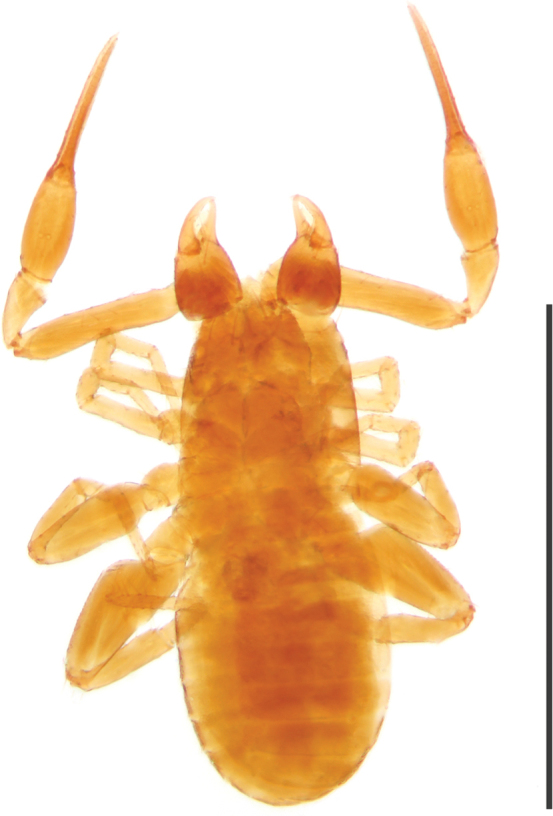
*Tyrannochthonius eua* sp. nov., holotype male, dorsal. Scale bar: 1 mm.

***Dimensions*** (length/width or, in the case of the legs, length/depth) in mm. Body length 1.10. Pedipalp: trochanter 0.13/0.09, femur 0.37/0.09, patella 0.19/0.09, chela 0.57/0.11, hand 0.24/0.11, fixed finger 0.31, moveable finger 0.33. Chelicera 0.26/0.17, moveable finger 0.15. Carapace 0.38/0.39. Leg I: trochanter 0.09/0.08, femur 0.22/0.05, patella 0.11/0.04, tibia 0.13/0.04, tarsus 0.17/0.03. Leg IV: trochanter 0.11/0.09, femoropatella 0.32/0.17, tibia 0.24/0.07, metatarsus 0.12/0.06, tarsus 0.20/0.03.

##### Etymology.

The specific epithet refers to the island of Eua, on which this species occurs. It is used as a noun in apposition.

##### Remarks.

The presence of intercalary teeth on the fixed chelal finger but not on the moveable chelal finger is unusual in *Tyrannochthonius* species. However, a few other species possess this combination: *T. convivus* Beier, 1974, *T. brasiliensis* Mahnert, 1979, *T. amazonicus* Mahnert, 1979, *T. rex* Harvey, 1989, and *T. swiftae* Muchmore, 1993 (Beier 1974; Mahnert 1979; Harvey 1989; Muchmore 2000). *Tyrannochthonius eua* sp. nov. differs from *T. convivus*, *T. amazonicus*, *T. rex*, and *T. swiftae* by the significantly shorter palpal femur length (0.37 mm, versus 0.42–0.49 mm in *T. convivus*, 0.46–0.56 mm in *T. amazonicus*, 1.24–1.34 mm in *T. rex*, and 0.53 mm in *T. swiftae*). It also differs from *T. amazonicus*, *T. rex*, and *T. swiftae* in having a lower number of teeth on fixed chelal fingers. In contrast, *T. brasiliensis* has a shorter palpal femur (length 0.28 mm) than *T. eua* sp. nov., as well as lower number of coxal spines on coxae II (4–5, versus 8 in *T. eua* sp. nov.).

**Figure 3.**
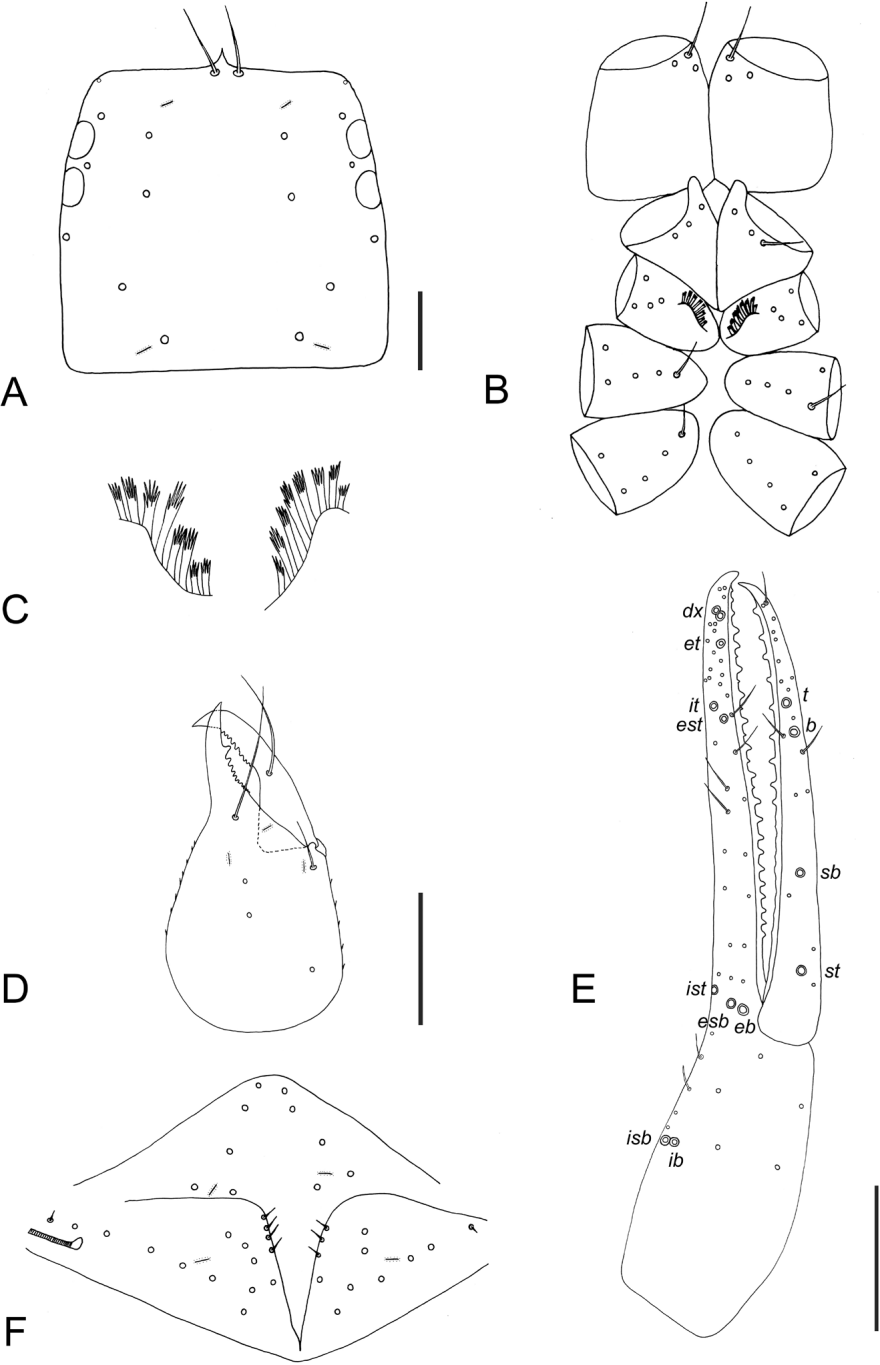
*Tyrannochthonius eua* sp. nov., holotype male, dorsal **A** carapace **B** coxae **C** coxal spines **D** right chelicera **E** right chela, showing trichobothrial pattern **F** chaetotaxy of genital area (sternites II–III). Abbreviations: trichobothria of moveable chelal finger: *t*–terminal, *b*–basal, *sb*–subbasal, *st*–subterminal; trichobothria of fixed chelal finger: *dx*–duplex trichobothria, *et*–exterior terminal, *it*–interior terminal, *est*–exterior subterminal, *ist*–interior subterminal, *esb*–exterior subbasal, *eb*–exterior basal, *isb*–interior subbasal, *ib*–interior basal. Scale bars: 0.1 mm.

### Cheiridiidae Hansen, 1894

#### Cheiridiinae Hansen, 1894

##### Nesocheiridium

Taxon classificationAnimaliaPseudoscorpionesCheiridiidae

Beier, 1957

1748BCAE-FD11-5AED-AC97-BF9C45F0408F

###### Diagnosis.

Small species, with adult body length ranging from 0.85 to 0.94 mm. Integument coarsely granulate, dorsally granuloreticulate. Vestitural setae relatively long, arcuate with a small spine, often covered by a fine exudate, giving them a leaf-like shape. Carapace narrowed towards anterior end, with short cucullus and a deep, submedian, transverse furrow. One pair of eyes. Cheliceral hand with four setae (seta *ls* absent), all acuminate. Galea long and slender, simple in male, with three terminal rami in female. Rallum of four blades, distal one enlarged and dentate. Ten abdominal tergites visible in dorsal view, I–IX divided. Ventral anal opening large and circular. Pedipalps densely and strongly granulate, including hand and the base of the fixed fingers, femur pedicellate. Fixed chelal finger with granulate swelling mesally, most distinct from ventro-lateral view. Chelal fingers slightly shorter than hand without pedicel. Venom apparatus present in both chelal fingers. Seven trichobothria present on fixed chelal finger, situated mainly in its basal half. Trichobothria *ib* and *ist* located distad of the granulate swelling, *eb* and *esb* situated close together subbasally. Moveable chelal finger with two trichobothria, situated in its basal half.

###### Remarks.

*Nesocheiridium* shares a combination of characters with most genera in the subfamily Cheiridiinae: reduced number of trichobothria on fixed chelal finger (seven at most) and moveable finger (two at most), four setae present on cheliceral hand, first blade of rallum enlarged, femur and patella of legs fused, tarsus of legs as about the same length as tibia (Chamberlin 1931; Beier 1957). The present study confirms the characters mentioned by Beier (1957) to justify the genus *Nesocheiridium*, namely the short cucullus, presence of a granulate swelling on the fixed chelal finger, trichobothria *ib* and *ist* located distad of the granulate swelling, and *eb* and *esb* situated close together subbasally.

##### Nesocheiridium stellatum

Taxon classificationAnimaliaPseudoscorpionesCheiridiidae

Beier, 1957

5B6AF392-D24D-543D-86F9-60A20685DD9F

Figs 1 , 4A , 5

###### Material examined.

***Holotype***: Northern Mariana Islands • ♂; Saipan, Mount Marpi [15.283, 145.817]; 40 m a.s.l.; 01 Mar. 1945; Henry S. Dybas leg.; under stone; **FMNH-INS** 0000 011 070.

###### Redescription.

**Figure 4.**
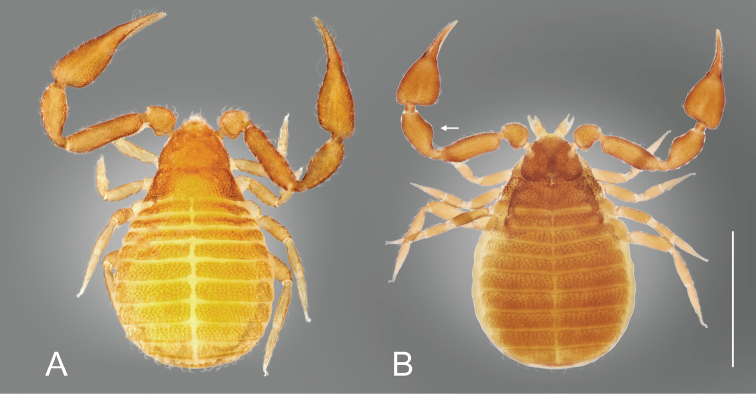
*Nesocheiridium* species, dorsal view **A***N. stellatum*, holotype male **B***N. onevai* sp. nov., holotype female. Arrow indicates widening of palpal patella. Scale bar: 0.5 mm.

**Figure 5.**
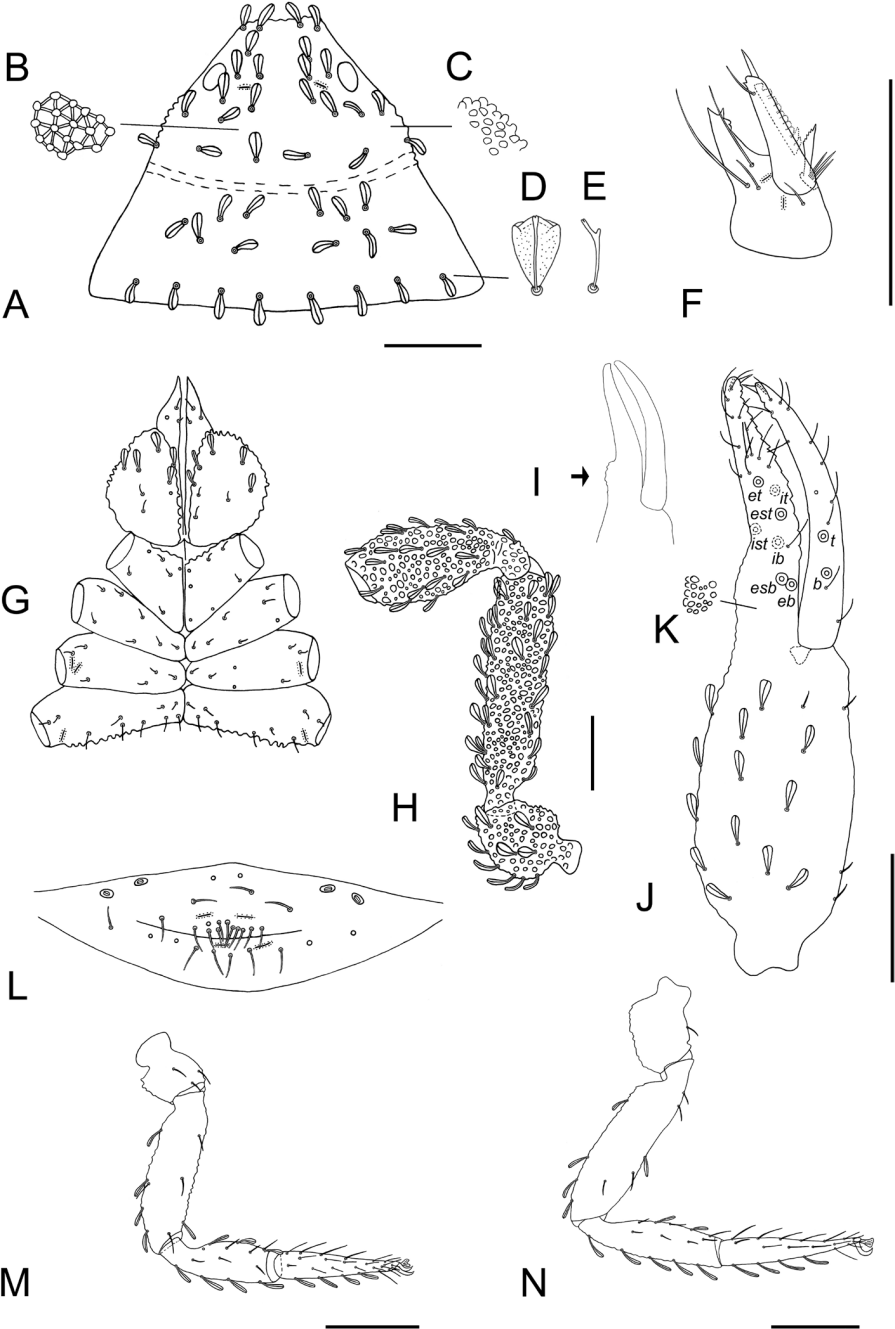
*Nesocheiridium stellatum*, holotype male **A** carapace **B, C** details of granulation types on carapace **D** leaf-like seta **E** arcuate seta with a small spine **F** right chelicera **G** coxae **H** right palp minus chela **I** chelal fingers, ventro-lateral view, showing swelling on fixed finger **J** right chela **K** detail of granulation on chela **L** chaetotaxy of genital area (sternites II–III) **M** right leg I **N** right leg IV. Abbreviations as for Figure 3. Scale bars: 0.1 mm.

***Adult male*** (Figs 4A, 5). Integument coarsely granulate, dorsally granuloreticulate (Fig. 5B, C). Vestitural setae arcuate with a small spine, often covered by a fine exudate giving them a leaf-like shape (Fig. 5A, D, E). ***Carapace*** (Figs 4A, 5A): 0.85 × longer than broad, subtriangular, distally narrowed; cucullus short; two distinct eyes with lenses; submedian transverse furrow deep; anterior disk laterally with perceptible swellings; posterior disk without a medial depression (Fig. 4A); with 43 leaf-like setae (24 before furrow, 19 behind); with one pair of lyrifissures in ocular area. ***Chelicera*** (Fig. 5F): 1.60 × longer than broad; four setae on hand, all setae acuminate; moveable finger with one short seta; fixed finger with three teeth near the tip; with two lyrifissures on hand; galea long, slender, stylet-like, without rami; serrula exterior with 10 blades; rallum consisting of four blades. ***Coxae*** (Fig. 5G): coarsely granulate; chaetotaxy: manducatory process three acuminate setae, rest of palpal coxa with three acuminate and five leaf-like setae in anterior half; pedal coxae I six or seven acuminate setae, II seven acuminate setae, III six or seven acuminate setae, IV 9 or 10 acuminate setae. Lyrifissures: one or two on coxa III, 1 on coxa IV; maxillary lyrifissures not visible. ***Pedipalp*** (Fig. 5H–K): coarsely granulate; trochanter with distinct dorsal hump; patella with distinct pedicel. Trochanter 1.40 ×, femur 4.13 ×, patella 2.89 ×, chela 3.62 ×, hand with pedicel 2.15 × longer than broad. Chela, including base of fixed finger, coarsely granulate (Fig. 5K). Venom apparatus present in both fingers. Fixed chelal finger with seven trichobothria, moveable finger with two. Fixed finger with 16 flat marginal teeth; moveable finger with three flat marginal teeth. Fixed finger with granulate swelling mesally (Fig. 5I), trichobothria *ib* and *ist* distad of swelling. Arrangement of trichobothria as in Figure 5J. ***Opisthosoma***: Tergal chaetotaxy: 4+5: 6+6: 6+6: 6+7: 7+6: 7+7: 8+8: 7+7: 6+5: 5+5; I–X with leaf-like setae. Tergal lyrifissures I–X: 0+0: 1+1: 1+1: 1+1: 1+1: 1+1: 1+1: 0+0: 0+0: 0+0: 0+0. Tergal pores I–X: 0+0: 0+0: 0+0: 0+0: 2+2: 0+0: 2+1: 0+0: 0+3: 0+3. Sternal chaetotaxy (Fig. 5L): 17: 10: 5+4: 7+6: 7+8: 7+7: 5+6: 7+6: 5+6: 3+3; II–VII with acuminate setae, VIII–XI with leaf-like setae. Sternal lyrifissures II–XI: 2: 2: 1+1: 0+0: 1+1: 1+1: 1+1: 1+1: 1+1: 0+0. Sternal pores II–XI: 4: 0: 7+8: 7+7: 5+6: 1+1: 0+0: 0+0: 1+2: 3+3. Anal opercula each with two short, acuminate setae. Genitalia not studied in detail. ***Leg I*** (Fig. 5M): trochanter 0.86 ×, femoropatella 3.60 ×, tibia 3.00 ×, tarsus 4.33 × deeper than broad. ***Leg IV*** (Fig. 5N): trochanter 1.43 ×, femoropatella 3.83 ×, tibia 3.60 ×, tarsus 5.67 × longer than deep. No tactile setae present; claws simple; arolia shorter than claws.

***Dimensions*** (length/width or, for legs, length/depth) in mm. Body length 0.94. Pedipalp: trochanter 0.14/0.10, femur 0.33/0.08, patella 0.26/0.09, chela 0.47/0.13, hand with pedicel 0.28/0.13, hand without pedicel 0.23, moveable finger 0.21. Chelicera: 0.08/0.05, moveable finger 0.07. Carapace 0.34/0.40. Leg I: trochanter 0.06/0.07, femoropatella 0.18/0.05, tibia 0.12/0.04, tarsus 0.13/0.03. Leg IV: trochanter 0.10/0.07, femoropatella 0.23/0.06, tibia 0.18/0.05, tarsus 0.17/0.03.

###### Remarks.

Some of the morphometric values given here differ slightly from the original description (Beier 1957) (e.g., body size 0.94 versus 0.90 mm; length of carapace 0.34 versus 0.32 mm; width of carapace 0.40 versus 0.37 mm).

##### Nesocheiridium onevai sp. nov.

Taxon classificationAnimaliaPseudoscorpionesCheiridiidae

0D961D1C-82E5-516A-81EE-8530A05BF590

http://zoobank.org/1EBB04B6-8414-42BE-950D-BE50D4397D44

Figs 1 , 4B , 6

###### Material examined.

***Holotype***: Polynesia • ♀; Tonga, Onevai [-21.087, -175.115]; 7 m a.s.l.; 10 Jun. 1980; Galina Fedorovna Kurcheva leg.; moss; **NHMW** 29188.

###### Description.

**Figure 6.**
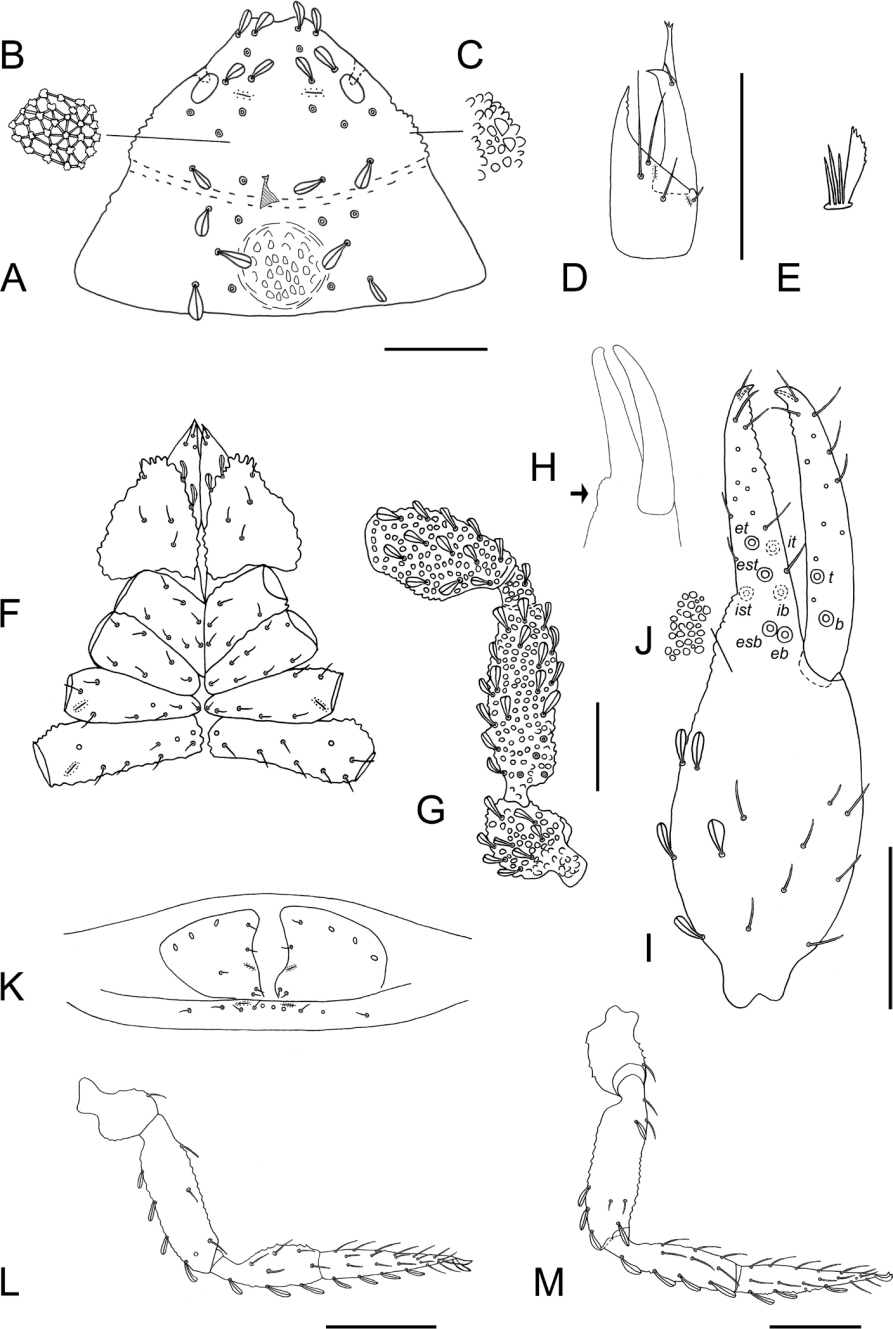
*Nesocheiridium onevai* sp. nov., holotype female **A** carapace (damaged part cross-hatched) **B, C** details of granulation types on carapace **D** right chelicera **E** rallum **F** coxae **G** right palp minus chela **H** chelal fingers, ventro-lateral view, showing swelling on fixed finger **I** right chela **J** detail of granulation on chela **K** chaetotaxy of genital area (sternites II–III) **L** right leg I **M** right leg IV. Abbreviations as for Figure 3. Scale bars: 0.1 mm.

***Adult female*** (Figs 4B, 6). Integument coarsely granulate, dorsally granuloreticulate (Fig. 6B, C). Vestitural setae arcuate with a small spine, often covered by a fine exudate, giving them a leaf-like shape. ***Carapace*** (Fig. 6A): 0.72 × longer than broad, subtriangular; cucullus short; two distinct eyes with lenses; two lateral lighter sections at the level of the eyes (this is not due to damage); submedian transverse furrow deep (carapace slightly damaged in middle); anterior disk laterally with two protuberances, posterior disk with a shallow medial depression in its middle (Fig. 6A); with 30 leaf-like setae (20 before furrow, 10 behind); with one pair of lyrifissures in ocular area. ***Chelicera*** (Fig. 6D): 1.80 × longer than broad; four setae on hand, all acuminate; moveable finger with one short seta; fixed finger with two teeth near tip; with two lyrifissures on hand; galea long, slender, with three apical rami; serrula exterior with 10 blades; rallum consisting of four blades (Fig. 6E). ***Coxae*** (Fig. 6F): coarsely granulate; chaetotaxy: manducatory process two or three acuminate setae, rest of palpal coxa with four acuminate and two leaf-like setae, situated in anterior half; pedal coxae I 5–7 acuminate setae, II 6–8 acuminate setae, III seven or eight acuminate setae, IV eight or nine acuminate setae. Lyrifissures: one on coxa III, one on coxa IV; maxillary lyrifissures not visible.

***Pedipalp*** (Fig. 6G–J): coarsely granulate; trochanter with distinct dorsal hump; patella markedly broadened mesally, with a distinct pedicel (Fig. 4B). Trochanter 1.56 ×, femur 3.25 ×, patella 2.10 ×, chela 2.60 ×, hand with pedicel 1.40 × longer than broad. Chela, including the base of the fixed finger, coarsely granulate (Fig. 6J). Venom apparatus present in both fingers. Fixed chelal finger with seven trichobothria, moveable chelal finger with two trichobothria. Fixed chelal finger with 10 flat marginal teeth; moveable finger with four flat marginal teeth. Fixed finger with granulate swelling mesally (Fig. 6H), trichobothria *ib* and *ist* distad of swelling. Trichobothrial pattern as in Figure 6I. ***Opisthosoma***: Tergal chaetotaxy: 3+4: 4+4: 5+5: 6+7: 7+7: 7+7: 6+7: 7+6: 5+5: 4+3; I–X with leaf-like setae. Tergites without lyrifissures. Tergal pores I–X: 0+0: 1+2: 1+2: 2+2: 1+2: 2+2: 1+2: 2+2: 0+0: 0+0. Sternal chaetotaxy (Fig. 6K): 5+4 small entrance setae: 10: 4+5: 6+6: 6+7: 7+7: 7+7: 7+7: 5+4: 3+3; II–VIII with acuminate setae, IX–XI with leaf-like setae. Sternal lyrifissures II–XI: 1+1: 2: 0+0: 1+2: 1+1: 1+1: 1+1: 1+1: 1+1: 0+0. Sternal pores II–XI: 6: 0: 5+5: 5+5: 5+4: 0+1: 0+0: 0+0: 1+1: 3+4. Anal opercula: each with two short, acuminate setae. Anterior genital operculum with sternal plates divided. Genitalia not studied in detail. ***Leg I*** (Fig. 6L): trochanter 1.00 ×, femoropatella 3.00 ×, tibia 2.75 ×, tarsus 4.33 × longer than deep. ***Leg IV*** (Fig. 6M): trochanter 1.67 ×, femoropatella 3.80 ×, tibia 3.75 ×, tarsus 5.33 × longer than deep. No tactile setae present; claws simple.

***Dimensions*** (length/width or, for legs, length/depth) in (mm). Body length 0.85. Pedipalp: trochanter 0.14/0.09, femur 0.26/0.08, patella 0.21/0.10, chela 0.39/0.15, hand with pedicel 0.21/0.15, hand without pedicel 0.19, moveable finger 0.18. Chelicera: 0.09/0.05, moveable finger 0.07. Carapace 0.28/0.39. Leg I: trochanter 0.05/0.05, femoropatella 0.15/0.05, tibia 0.11/0.04, tarsus 0.13/0.03. Leg IV: trochanter 0.10/0.06, femoropatella 0.19/0.05, tibia 0.15/0.04, tarsus 0.16/0.03.

###### Etymology.

The species epithet refers to the island Onevai, on which this species occurs. It is used as a noun in apposition.

###### Remarks.

The two species currently placed in the genus are easy to distinguish from each other by the form of the carapace (*N. stellatum* lacks a medial depression on the posterior disk, whereas *N. onevai* sp. nov. has a weak medial depression on the posterior disk); the shape of the palpal patella (not broadened in *N. stellatum*, versus markedly broadened mesally in *N. onevai* sp. nov.); the number of setae on the carapace (43 in *N. stellatum*, 30 in *N. onevai* sp. nov.); the number of marginal teeth on the fixed chelal finger (16 in *N. stellatum*, 10 in *N. onevai* sp. nov.); the shape of the setae on sternite VIII (leaf-like in *N. stellatum*, as opposed to acuminate in *N. onevai* sp. nov.); and the lengths of the palpal segments (femur 0.33 mm in *N. stellatum*, 0.26 mm in *N. onevai* sp. nov.; patella 0.26 mm in *N. stellatum*, 0.21 mm in *N. onevai* sp. nov.; hand with pedicel 0.28 mm in *N. stellatum*, 0.21 mm in *N. onevai* sp. nov.).

## Discussion

The original description of the genus *Nesocheiridium* was based on a single male of *N. stellatum*. The discovery of a new, congeneric species affords the opportunity to clarify the diagnostic characters of this inadequately known genus. The presence of a granulate swelling on the base of the fixed chelal finger is considered to be the main diagnostic character of *Nesocheiridium*. However, it is worth noting that, although this character has not previously been mentioned in descriptions of other Cheiridiinae, some drawings, such as those published for *Neocheiridium corticum* (Balzan, 1877) by Mahnert and Aguiar (1986) and for *N. africanum* Mahnert, 1982 by Mahnert (1982), indicate its presence in *Neocheiridium*. Thus, despite a better understanding of the species of *Nesocheiridium*, doubts remain about the validity of the genus. One obstacle to clearly defining the genus within Cheiridiinae is the fact that many of the other genera of Cheiridiinae remain inadequately diagnosed. The need for revisionary work in this subfamily Cheiridiinae has previously been mentioned by other authors (e.g., Mahnert and Aguiar 1986; Judson 2000; Sammet et al. 2020).

## Supplementary Material

XML Treatment for Tyrannochthonius

XML Treatment for Tyrannochthonius eua

XML Treatment for Nesocheiridium

XML Treatment for Nesocheiridium stellatum

XML Treatment for Nesocheiridium onevai
